# Plasmobase: a comparative database of predicted domain architectures for *Plasmodium* genomes

**DOI:** 10.1186/s12936-017-1887-8

**Published:** 2017-06-07

**Authors:** Juliana Bernardes, Catherine Vaquero, Alessandra Carbone

**Affiliations:** 1 Laboratoire de Biologie Computationnelle et Quantitative, UMR 7238, IBPS, CNRS, UPMC Univ-Paris 6, Sorbonne Universités, 4 place Jussieu, 75005 Paris, France; 2 Centre d’ Immunologie et des Maladies Infectieuses (CIMI-Paris), CNRS ERL 8255, INSERM U1135, UPMC Univ-Paris 6, Sorbonne Universités, Paris, France; 3 Institut Universitaire de France, 75005 Paris, France

**Keywords:** Plasmodium, Genome, Protein architecture, Domain architecture prediction, Annotation, Genome comparison, Database

## Abstract

**Background:**

With the availability of complete genome sequences of both human and non-human *Plasmodium* parasites, it is now possible to use comparative genomics to look for orthology across *Plasmodium* species and for species specific genes. This comparative analyses could provide important clues for the development of new strategies to prevent and treat malaria in humans, however, the number of functionally annotated proteins is still low for all *Plasmodium* species. In the context of genomes that are hard to annotate because of sequence divergence, such as *Plasmodium*, domain co-occurrence becomes particularly important to trust predictions. In particular, domain architecture prediction can be used to improve the performance of existing annotation methods since homologous proteins might share their architectural context.

**Results:**

Plasmobase is a unique database designed for the comparative study of *Plasmodium* genomes. Domain architecture reconstruction in Plasmobase relies on DAMA, the state-of-the-art method in architecture prediction, while domain annotation is realised with CLADE, a novel annotation tool based on a multi-source strategy. Plasmobase significantly increases the Pfam domain coverage of all *Plasmodium* genomes, it proposes new domain architectures as well as new domain families that have never been reported before for these genomes. It proposes a visualization of domain architectures and allows for an easy comparison among architectures within *Plasmodium* species and with other species, described in UniProt.

**Conclusions:**

Plasmobase is a valuable new resource for domain annotation in *Plasmodium* genomes. Its graphical presentation of protein sequences, based on domain architectures, will hopefully be of interest for comparative genomic studies. It should help to discover species-specific genes, possibly underlying important phenotypic differences between parasites, and orthologous gene families for deciphering the biology of these complex and important Apicomplexan organisms. In conclusion, Plasmobase is a flexible and rich site where any biologist can find something of his/her own interest.

**Availability:**

Plasmobase is accessible at http://genome.lcqb.upmc.fr/plasmobase/.

## Background

A large amount of genomic and post-genomic data is now available for the *Plasmodium* genus. The fully sequenced genomes of 11 species are accessible from PlasmoDB [1], but despite the availability of these complete genomes a major limitation still remains on their functional annotation. The number of proteins with unknown functions is still high for all *Plasmodium* species. This could be due (i) to the AT-richness of these genomes, (ii) to the specificity of a number of parasitic functional mechanisms evolved to evade host immune recognition, (iii) to the strong divergence of *Plasmodium* protein sequences that make homology detection a difficult task. These three reasons might have contributed to the development of new gene functions and changes in the parasite’s genome, occurring through gene acquisition and deletion [2].

Proteins are composed of one or more “domains”, that is structural motifs that can evolve, function, and exist independently of the rest of the protein chain. Domains might be found in different combinations, and the arrangement of these domains in a protein forms the so called “protein architecture”. By focalizing on domain recognition, genome annotation can be highly improved. Several approaches and databases have been developed to detect and identify functional domains in a large number of proteins, including Pfam [3], SMART [4], and PROSITE [5]. Based on these resources, a number of conflicting domain predictions (potential domains) can be resolved and, for each protein to be annotated, a domain architecture can be proposed. There are different methods for identifying a domain architecture and the most successful ones explore domain co-occurrence for controlling the false discovery rate (FDR) associated with the predictions [6–8]. Here, DAMA [8] (domain annotation by a multi-objective approach), an approach that treats protein domain architecture prediction as a multi-objective optimization problem, is used. DAMA combines a number of criteria to handle multi-(possibly pairwise-) domain co-occurrence and domain overlapping, and it outperforms existing methods. It detects domain architectures with a larger number of domain co-occurrences.

DAMA can improve domain recognition methods such as HMMer [9, 10] used by Pfam, but it is limited by the number of potential domains given as input. Hence, CLADE (closer sequences for annotations directed by evolution) [11], the new generation of annotation tools based on a “multi-source strategy”, is used to increase the set of potential domains and consequently to improve DAMA performance. CLADE uses several hundred probabilistic profiles to represent each Pfam domain instead of one profile, based on global consensus, as for mono-source strategies. These probabilistic models are originated from different species, spanning the whole phylogenetic tree, and describe alternative evolutionary pathways for a domain. Tested on the *Plasmodium falciparum* genome, CLADE outperforms the widely used tools based on a mono-source annotation strategy, HMMer and HHblits [12]. The new domain annotation obtained by CLADE for *P. falciparum 3D7* has been released with [11].

Plasmobase, presented here, is a novel database reporting known and new protein domains identified by DAMA and CLADE on the 11 fully sequenced genomes in PlasmoDB (see Table 1). Plasmobase contains a large number of newly discovered domains in each *Plasmodium* genome, leading to an enrichment of 18–30% of the total number of domain families when compared to Pfam predictions (with an FDR <1%). In addition, Plasmobase is a unique platform for the comparative study of *Plasmodium* genomes. It proposes a visualization of domain architectures and it allows for an easy comparison among architectures within *Plasmodium* species and all other species in UniProt. A friendly interface allows users to interact with the platform to access new annotations and possibly detect annotation errors.

**Table 1:** Features of *Plasmodium* genomes

Species	Genome size (Mb)	#Proteins^a^	AT%^b^
*P. falciparum 3D7*	23.33	5542	0.81
*P. falciparum IT*	22.98	5491	0.81
*P. vivax*	27.01	5586	0.58
*P. knowlesi H*	24.40	5229	0.61
*P. cynomolgi*	26.18	5716	0.60
*P. reichenowi CDC*	23.92	5846	0.81
*P. chabaudi*	18.97	5217	0.76
*P. berghei*	18.78	5076	0.78
*P. yoelii 17X*	22.76	5978	0.78
*P. yoelii 17XNL*	22.94	7724	0.76
*P. yoelii YM*	22.03	5709	0.78

Numbers are reported from PlasmoDB [1]

^a^The symbol # stands for “the number of”

^b^AT richness is computed on CDS regions only

## Methods

### Data

#### Plasmodium species

The 11 fully sequenced genomes present in PlasmoDB [1] and considered here are: three human parasites (*P. falciparum 3D7*, *P. falciparum IT*, and *Plasmodium vivax*), two macaques parasites (*Plasmodium knowlesi* and *Plasmodium cynomolgi*), one chimpanzee parasite (*Plasmodium reichenowi*), and five rodent parasites (*Plasmodium chabaudi*, *Plasmodium berghei*, and *Plasmodium yoelii* *17X*, *Plasmodium yoelii yoelii 17XNL* and *Plasmodium yoelii YM*). All genomes were extracted from http://PlasmoDB.org, the official repository of the *Plasmodium* proteins used as a reference database by malaria researchers. For each species, the genome size and the number of proteins are shown in Table 1.

#### The UniProt database

In order to display the proportion of proteins with similar architecture to a given query protein, all proteins (18,523,877) with known Pfam domain architectures are extracted from UniProtKB [13] and organized according to their taxon groups. Four taxon groups are considered: Eukaryota, Bacteria, Archaea and “Viruses and others” including metagenome and unclassified sequences. These architectures are obtained by parsing the file swisspfam, available at ftp://ftp.ebi.ac.uk/pub/databases/Pfam/releases/Pfam27.0/swisspfam.gz. Within each taxon group, sequences are grouped according to the name of their phylogenetic clade. A generic clade named “others” collects all clades with less than 2% of domain architectures. A reference list of clades has been extracted from NCBI (http://www.ncbi.nlm.nih.gov/taxonomy) and used for organizing the species where similar architectures are found.

#### GO terms

The gene ontology initiative [14], besides its value as a database of annotations, maintains and develops a controlled vocabulary of gene and gene product attributes. The gene ontology terms (GO terms) [15] is a machine-readable vocabulary that provides a standard output for functional predictions, avoiding the ambiguity of natural language. GO terms describe three aspects of gene product function: molecular function, biological process, and cellular location. pfam2go [16], a mapping that associates a specific GO term with a Pfam domain, is used to provide GO terms for Plasmobase domain predictions. In consequence, all proteins containing this domain share the same GO term.

### Tools

CLADE [11] and DAMA [8] were applied to all *Plasmodium* organisms, CLADE to identify potential domains and DAMA to reconstruct protein domain architectures. Both tools were run with default parameters, corresponding to a FDR smaller than 1%. The two tools are briefly described below. For the FDR estimation and other details refer to the original articles.

#### CLADE: closer sequences for annotations directed by evolution

CLADE is a computational approach that highly increases the sensitivity of domain prediction. It is a multi-source approach where several hundred probabilistic profiles are used to represent each domain, instead of one as for mono-source strategies, employed by methods like HMMer [9, 10] and HHblits [12]. CLADE predicts domains based on two classes of probabilistic profiles. The first is the profile library available in the Pfam database (version 27). There are 14,831 profiles, one for each domain. These profiles capture the consensus of homologous sequences, and the idea behind them is that homologous proteins should share common physico-chemical and structural features that could be described by consensus on the entire set of homologs.

The second class of profiles is constituted by hundreds of probabilistic models, associated to each Pfam domain. They are constructed starting from homologous sequences spreading a large panel of taxonomic origins, to guarantee that the phylogenetic tree is well represented. For each selected homologous sequence, a specific profile, named “clade-centred model” (CCM), is constructed. To construct a CCM, the selected homologous sequence is used as a query to search for close homologs within the non-redundant protein database (NR) with PSI-BLAST [17]. The CCM is the resulting PSI-BLAST profile. Note that PSI-BLAST constructs the CCM profile from NR sequences detected with an E-value threshold set to 1*e*−3 by default. CLADE uses this PSI-BLAST default E-value (1*e*−3) and sets the number of PSI-BLAST iterations to 5. On average 161 CCMs were constructed to represent each Pfam domain. These models span regions of protein sequence space that are not well represented by Pfam consensus models, and they highlight motifs, structural characteristics or physico-chemical properties that are shared by similar homologous sequences. Hence, if the original set of sequences for a domain is made of very divergent homologs, clade-centred models, are expected to describe properties that could be missed by the original Pfam model, representing the global consensus.

The CLADE library, made of Pfam consensus models and clade-centred models, is composed of 2,404,066 profiles. This library is used to identify potential domains in query proteins. Potential domains are then filtered by using support vector machines (SVM) combining various optimisation criteria, ultimately converted into a score, to specifically deal with false positives and discriminate domains. The SVM selects most probable predictions among domain hits displaying a small E-value, a sufficiently long domain hit, the phylogenetic proximity between the taxon of the sequence to be annotated and the reference species generating the probabilistic profile, and the agreement among models leading to the prediction. This filtering step is fundamental in domain prediction. Once domains are filtered, CLADE calls DAMA to find the most probable architecture for a given query sequence. CLADE can be downloaded at http://www.lcqb.upmc.fr/CLADE.

#### DAMA: domain annotation by a multi-objective approach

Since homologous proteins might share their architectural context, domain architecture prediction are used to improve the performance of CLADE annotation. The problem can be complex when a query sequence matches several probabilistic models, producing a set of conflicting predictions with overlapping domain boundaries. To address this problem, DAMA combines a number of criteria including multi- (possibly pairwise-) domain co-occurrence and domain overlapping. Domain co-occurrence is expected to enhance the level of confidence in a prediction [18] mainly because (i) the majority of proteins are multi-domain and (ii) fewer combinations than the statistically expected ones are observed. Some overlapping must also be admitted to increase the number of correct domain predictions, as demonstrated in [19]. DAMA encodes domain co-occurrence and hit overlapping criteria into objective functions, and treats protein domain architecture prediction as a multi-objective optimization problem. First, DAMA generates a list of possible architectures, and then maximizes a set of objective functions to select the best architecture. Five functions, designed according to several objectives, are applied in order of importance. The first objective function ensures that higher confidence domains are in the final architecture since domain scores greatly augment the trust on protein annotation. The second and the third function explore the tendency of some domain families to occur preferentially with a few other favourite families. The second function maximizes the number of multi-domain co-occurrences, while the third one maximizes the number of pairwise domain combinations when two architectures present the same number of multi-domain co-occurrences. The fourth function privileges architectures with distinct domains since domain duplication is less likely to change the protein function. Finally, the fifth function selects the architecture whose domains have the highest scores. DAMA can be downloaded at http://www.lcqb.upmc.fr/DAMA.

DAMA was used with default parameters, including the parameter ‘- - review” that adds new domains into an architecture if: (1) they present a significant E-value (<1*e*−10) and (2) they do not overlap an existing domain in the architecture. By setting this parameter, one can increase the number of predicted domains and takes into account the identification of new domain architectures (see "Discussion").

### Data availability

Plasmobase website provides access to downloadable xls files containing the full list of annotations for the 11 *Plasmodium* species. Each file contains, for each domain hit, the PlasmoDB accession number of the sequence where the hit is identified, its starting and ending position, the Pfam domain name, the Pfam accession number, the E-value, CLADE SVM probability, the model identifier in the CLADE model library (either a clade-centred model or a Pfam consensus model), the start and the end position of the hit either in the domain sequence used to construct the clade-centred model or in the Pfam consensus model, the clade name (if any) and the organism name of the sequence originating the clade-centred model used for the domain identification. Note that if a protein sequence is annotated by several domains, the file contains several rows, one for each domain annotation.

## Results

Plasmobase is a platform for the exploration of protein architectures and their comparison across species. Its features and its multiple ways to analyse a predicted architecture or to explore potential architectures, are presented together with some global statistics on new domain annotations for the 11 *Plasmodium* genomes.

### The Plasmobase platform

The *Plasmodium* database Plasmobase is a friendly interface allowing users to search by domains (with a Pfam accession number or a Pfam description), proteins (with a protein accession number or a protein annotation) or domain architectures (with a list of Pfam accession numbers) on all *Plasmodium* species or on a specific one. As a result, it provides the list of corresponding proteins with their accession number, *Plasmodium* species name, PlasmoDB annotation, a list of domains forming the predicted architecture (where new domains are highlighted), and the accessibility (“Look up” link) to a graphical interface providing comparative information on the predicted architecture. Each protein in the list is linked to its PlasmoDB description, and each domain forming the protein architecture is linked to its Pfam description.

**Fig. 1:**
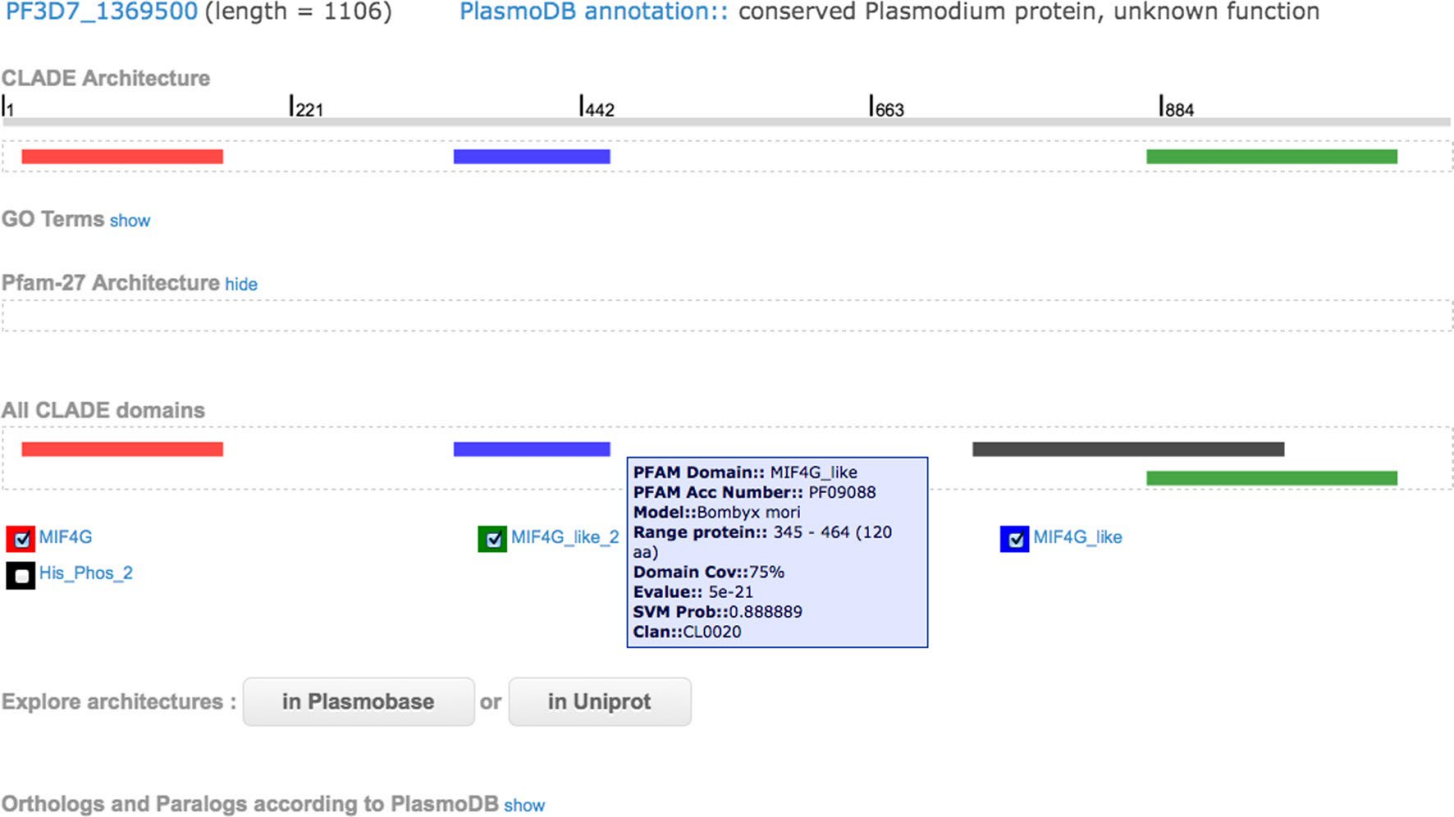
Newly predicted domain architecture of *P. falciparum *gene PF3D7_1369500. Plasmobase “Look up” page associated to gene PF3D7_1369500. CLADE predicted architecture (*top*) contains three domains: MIF4G, MIF4G_like_2, MIF4G_like. Pfam_27 architecture is displayed below and it highlights no identified domains. The list of all domains identified by CLADE is given. Besides the three domains belonging to CLADE architecture, there is one more domain displayed in *grey* that has been also identified by CLADE but not selected by DAMA. The user might be interested to consider it in view of a putative functional annotation of the protein. Indeed, he/she can select a combination of CLADE domains and explore it either in Plasmobase or in UniProt by clicking on the corresponding buttons (*bottom*). The list of orthologs and paralogs, according to PlasmoDB, can be displayed by clicking on the “show” link. Windows with informations on identified domains (Pfam domain name, Pfam accession number, CLADE model species, position of the domain in the protein, domain coverage, E-value, CLADE SVM probability, clan name if any) are accessible by passing the mouse above the domain location, as illustrated by the information box for the blue domain. Note that the clade-centred model generated by the *Bombix mori* MIF4G_like sequence is the one that obtained the best match with *Plasmodium* sequence PF3D7_1369500

Figure 1 illustrates the graphical interface obtained when querying for the *P. falciparum* protein PF3D7_1369500, identified by PlasmoDB as a “conserved *Plasmodium* protein, with no known function” and no predicted domain. It is accessible through the “Look up” link. On the top, the graphical interface shows the protein details: PlasmoDB accession number, protein length, and PlasmoDB text annotation. Then, it displays the predicted architecture constituted by three new domains (MIF4G, MIF4G_like_2 and MIF4G_like—see the CLADE architecture box), and the GO terms associated to the domains (accessible by clicking “GO Terms show”). For each predicted domain, there is an interactive legend (appearing when the cursor passes over the domain icon) with the description of the domain (Pfam domain, Pfam accession number, species generating the CLADE model identifying the domain, protein length, domain coverage, E-value, CLADE SVM probability, Pfam clan). The “Pfam-27 architecture” box shows the annotation proposed by the Pfam database version 27, where domains are predicted by HMMer and the architecture is obtained by employing a simple strategy that considers highest confidence domains without overlapping. For PF3D7_1369500 no domain was predicted by HMMer, and an empty box is shown. This display is accessible by clicking on the “show” link. (In Fig. 1, the button “hide” allows hiding the display). Orthologs and paralogs according to PlasmoDB can also be displayed by clicking on the “show” link. Note that all PF3D7_1369500 orthologs are identified by PlasmoDB as “conserved *Plasmodium* protein, unknown function” or “hypothetical protein”.

**Fig. 2:**
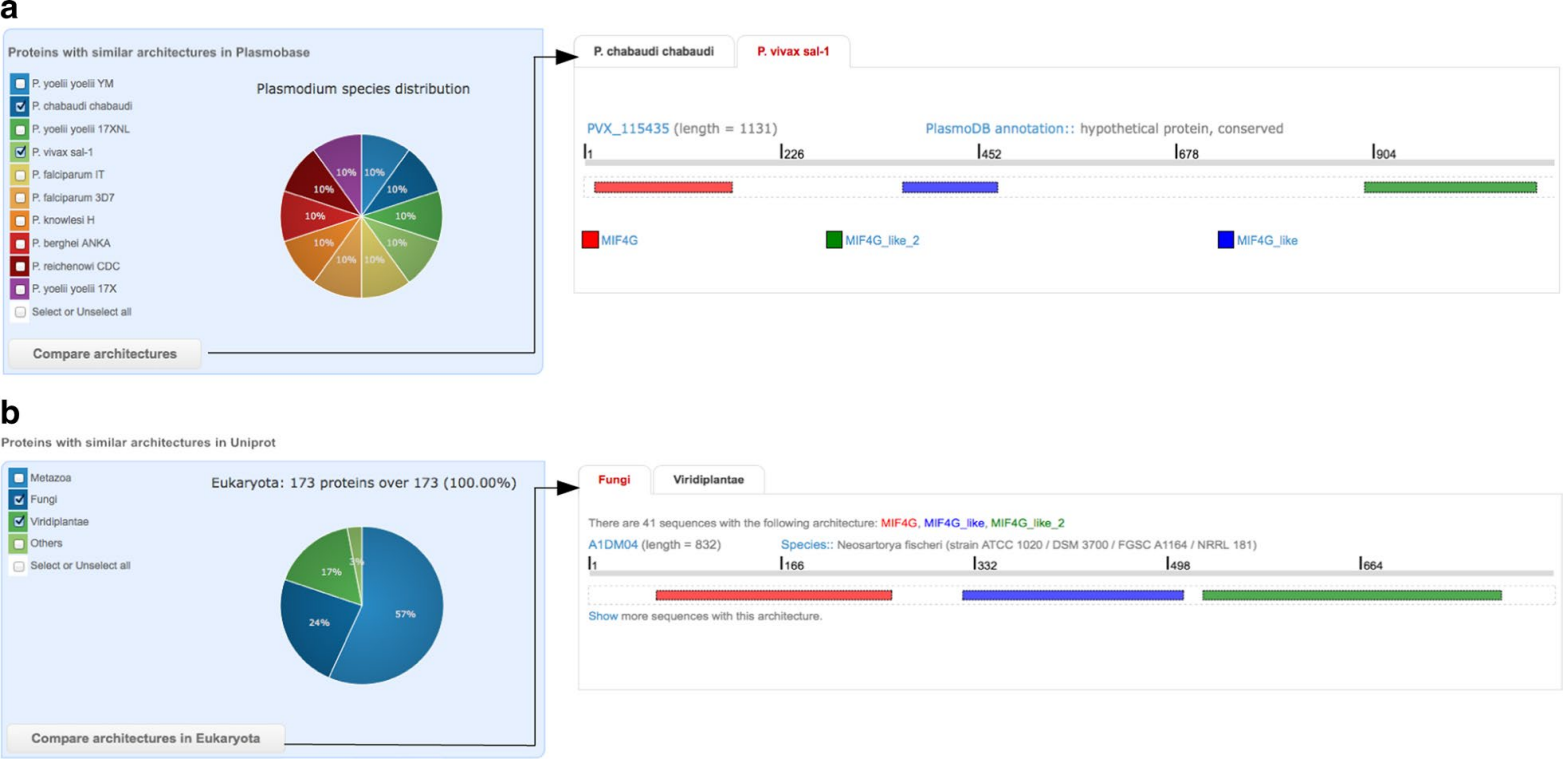
Proteins with similar architectures explored in Plasmobase/UniProt. **a** All *Plasmodium* species contain a protein sequence sharing the same CLADE architecture as PF3D7 1369500. A selection of these species allows to explore these protein sequences in Plasmobase, and verify information for domain architecture identification in other species. The whole list of domains is reported (in this specific example, there is only one architecture per species). **b** Plasmobase allows to explore the UniProt database for architectures that are similar to the one identified by CLADE for PF3D7 1369500. There are similar architectures in Metazoa, Fungi, Viridiplantae and other clades. A selection of Fungi and Viridiplantae allows the user to compare the architectures among these clades. Fungi contains 41 sequences with the given architecture and the full list is accessible

The list of all overlapping domains identified by CLADE, but not selected by DAMA to belong to the proposed architecture, is shown in the “All CLADE domains” box. For each domain in the list, those that are known to co-occur with it, are accessible by passing the mouse over the domain name. Plasmobase allows the user to evaluate the interest of an overlapping domain in view of a putative functional annotation for the protein. These domains can be explored in combination to other identified domains. In fact, the user can select any combination of CLADE domains (the ones belonging to the proposed CLADE architecture but also those that have been selected and filtered) and explore for their co-occurrence either in Plasmobase (Fig. 2a) or in UniProt (Fig. 2b) through the two dedicated button in the “Explore architectures” box (see below).

An immediate access to the list of architectures of orthologous and paralogous genes in *Plasmodium* species is possible by clicking on the “show” link at the bottom of the page (Fig. 1). Each protein accession number is provided together with the species name, the length of the sequence and the PlasmoDB annotation. The display of the associated Plasmobase architectures can be obtained by clicking the “Compare orthologous/paralogous architectures” button.

Figure 2a (left) shows all the *Plasmodium* species having protein sequences with a similar architecture to PF3D7_1369500 in Plasmobase. The proportion of proteins including the same co-occurring domains is shown by a pie chart. There are 10 orthologous proteins, in 10 *Plasmodium* species. The only exception is *P. cynomolgi* where MIF4G_like_2 was not identified. Architectures can be compared in more details by ticking some or all organisms and pressing the button “Compare architecture in Plasmobase”. In this example, *P. chabaudi* and *P. vivax* are selected, and the architectures are displayed in Fig. 2a (right), where CLADE domains are shown with the same graphical interface of PF3D7_1369500. Information on the species, functional annotation and GO classification is provided.

Figure 2b shows proteins with similar architecture in UniProt, the information is organized by taxon groups: Eukaryota, Bacteria, Archaea and “Viruses and others”. For PF3D7_1369500, 173 eukaryotic proteins with similar architectures and spread over several clades are found. The main clades (collecting most proteins) are shown in Fig. 2a (left) and the remaining ones are grouped in the checkbox “others” (see "Methods"). Like for Plasmobase, one can explore some or all clades and compare domain architectures for UniProt species within clades (button “Compare architecture in Eukaryota”). The architecture for Fungi proteins are shown in Fig. 2b (right). There are 41 proteins with the same architecture in Fungi and 29 in Viridiplantae. By clicking “Show”, all protein architectures are displayed.

### Many brand-new domains and many enriching ones

Those domains in Plasmobase that do not overlap PlasmoDB hits or that disagree with some PlasmoDB annotation are called “new”. Among new domains, there are some that are detected for the first time in the organism, and they are referred to as “brand-new” domains. The results are summarized in Table 2, where co-occurrent domains are highlighted (Cooc column) in regard to the total number of domains. For each species, the total number of new domains is shown in Table 2 (column “New domains”), this is the sum of domains that occur in proteins with no annotation in PlasmoDB (Table 2—column “First time”), and domains that enrich existing PlasmoDB architectures (Table 2—column “Enriching domains”). Note that more than half of the new domains enrich existing architectures and, interestingly, the rest provides an annotation of sequences that were never annotated before. The number of “brand-new” domains is reported in Table 2 (column “Brand new domains”). In average, each species gains more than 1500 new domains compared to existing PlasmoDB annotation, and two thirds of these domain predictions are supported by co-occurrence (see columns “Cooc” in Table 2abc), increasing their confidence.

**Table 2:** New domains identified in Plasmobase, possibly by co-occurrence (Cooc)

	First time^a^		Enriching domains^b^		New domains^c^		Brand-new domains^d^	
	Cooc^e^	Total^f^	Cooc^e^	Total^f^	Cooc^e^	Total^f^	Cooc^e^	Total^f^
*P. falciparum 3D7*	467	916	1052	1200	1519	2116	603	971
*P. falciparum IT*	368	691	893	984	1261	1675	496	741
*P. vivax*	324	659	871	996	1195	1655	525	824
*P. knowlesi H*	289	578	858	955	1147	1533	504	736
*P. cynomolgi*	296	614	703	792	999	1406	392	632
*P. reichenowi CDC*	373	679	911	1004	1284	1683	513	737
*P. chabaudi*	328	730	785	874	1113	1604	468	678
*P. berghei*	316	666	768	841	1084	1507	431	635
*P. yoelii 17X*	310	754	776	847	1086	1601	427	646
*P. yoelii yoelii 17XNL*	253	714	660	724	913	1438	370	556
*P. yoelii YM*	310	702	779	851	1089	1553	426	642

^a^Number of domain predictions occurring on proteins with no annotation in PlasmoDB

^b^Number of new domains enriching known protein architectures

^c^Total number of new domains, corresponding to the sum of a and b

^d^Number of new domains that occur in no proteins for the current *Plasmodium* species, according to PlasmoDB

^e^Number of predicted domains that are supported by co-occurrence

^f^Total number of identified domains, predicted or not based on co-occurrence

### Improvement over PlasmoDB

Table 3 compares the number of domains, found in each species, reported in PlasmoDB and Plasmobase. Plasmobase identifies a large number of Pfam domains in each *Plasmodium* species when compared to PlasmoDB predictions. These latter are based on HMMer (hmmscan) [9, 10] using the Pfam model library. The set of new predicted domains is expanded in all organisms, from 18% in *P. knowlesi H* to up to 30% in *P. falciparum 3D7*, and the number of proteins with no domain annotation largely decreased in all organisms. Compare the percentages of proteins with unknown function in Plasmobase and in PlasmoDB reported in Table 3.

**Table 3:** Comparison between PlasmoDB and Plasmobase domain predictions

Species	PlasmoDB		Plasmobase		
	#Pred domains	#Prots with no domain^a^	#Pred domains	%Improv^b^	#Prots with no domain^a^
*P. falciparum 3D7*	6037	2068 (37.31%)	7842	30	1526 (27.54%)
*P. falciparum IT*	5783	2085 (37.97%)	7035	22	1718 (31.29%)
*P. vivax*	5177	2132 (38.16%)	6431	24	1830 (32.76%)
*P. knowlesi H*	5469	1929 (36.89%)	6430	18	1627 (31.11%)
*P. cynomolgi*	4731	2449 (42.84%)	5660	20	2242 (39.22%)
*P. reichenowi CDC*	6110	2090 (35.75%)	7355	20	1734 (29.66%)
*P. chabaudi*	4834	2017 (38.66%)	6128	27	1572 (30.13%)
*P. berghei*	4715	1951 (38,43%)	5924	26	1566 (30.85%)
*P. yoelii 17X*	5564	2038 (34.09%)	6844	23	1872 (31.31%)
*P. yoelii yoelii 17XNL*	5134	4078 (52.79%)	6265	22	3311 (42.87%)
*P. yoelii YM*	5355	1981 (34.69%)	6592	23	1557 (27.27%)

^a^In parenthesis, the percentage of proteins with no domain is computed as #Prots with no domain/#Proteins, where #Proteins is reported in Table 1

^b^The improvement is computed as (#Predicted domains in Plasmobase—#Predicted domains in PlasmoDB)/#Predicted domains in Plasmobase

### Comparison with EuPathDomain

The EuPathDomain [20] is a protein domain database dedicated to ten eukaryotic human pathogens, including three *Plasmodium* species: *P. falciparum 3D7*, *P. vivax Sal-1* and *P. yoelii yoelii 17XNL*. It uses hmmscan with permissive E-values to generate a set of potential domains, CODD [6] is then used to predict domain architectures based on domain co-occurrence. Plasmobase improves over EuPathDomain in several manners. When compared with EuPathDomain, Plasmobase:

1. considers 11 *Plasmodium* species while EuPathDomain considers just 3 organisms,
2. proposes a visualization of the predicted architecture while EuPathDomain displays the list of predicted domains found within the sequence,
3. compares architectures in a species with architectures in other *Plasmodium* or in UniProt species, while EuPathDomain does not. Note that in Plasmobase, the user can explore domain architectures formed by any combination of CLADE domains, belonging to the proposed architecture or simply detected as potential hits of the sequence,
4. searches proteins by keywords (concerning both domains and protein functions, such as kinases, transcription, AP2 and HMGB, translation, 40S ribosomal) and not only by Pfam identifiers and genome accession numbers, as EuPathDomain,
5. allows searching for architectures described by multiple domains, while EuPathDomain only considers a single domain,
6. displays domain architectures for orthologous groups, while EuPathDomain does not.

Both systems provide GO functional annotation and, for each predicted domain, they indicate the other domains occurring in the architecture that are known to co-occur with it. Note that EuPathDomain displays Interpro domain predictions but it does not use Interpro database to predict new domains. Only Pfam domains are used, like in Plasmobase.

All information contained in the database can be downloaded in xls files, one for each species, permitting the user to process the information in alternative ways.

## Discussion

Protein annotation plays a major role in the comprehension of the biology of *Plasmodium* species. *Plasmodium* clade contains particularly AT rich genomes (Table 1) and this specificity of *Plasmodium* species makes Plasmobase contribution even more important. Indeed, Plasmobase provides a huge amount of new information concerning protein domain annotation across *Plasmodium* species. The new protein architectures suggested in Plasmobase can play a crucial role in the functional annotation of *Plasmodium* proteins, and potentially, on the identification of new functions, possibly rising from new domain combinations.

The reconstruction of the most likely domain architecture for a protein sequence constitutes one of the main steps of all predictive annotation strategies. Indeed, an accurate identification of the domain architecture of a multi-domain protein provides important information for function prediction, comparative genomics and molecular evolution. Here, the latest generation tools available (the new generation annotation method CLADE, employing a multi-source annotation strategy, and the state-of-the-art architecture reconstruction approach DAMA) are used to reconstruct the domain architecture of a large number of sequences presenting no domain annotation in Pfam_27 in order to annotate all *Plasmodium* complete genomes. The success of CLADE methodology was demonstrated on the *P. falciparum* genome [11]. Here, CLADE analysis is extended to 10 more species and a web interface simplifies the comparison between annotations by helping researchers to dig more profoundly into the evolution of the species and in the functional characteristics of their proteins. In Plasmobase, this can be done with a direct enquiry on functional keywords, associated to domains or proteins. Clearly, final confirmations are expected to be experimental.

*On false discovery rate in Plasmobase* Plasmobase provides multiple pieces of information to help users to evaluate a domain prediction: E-value, SVM probability, co-occurrences, and the possibility to compare predictions in orthologous genes. Indeed, one of the purposes of Plasmobase is to highlight several evidences to believe in the proposed domain annotation. Yet, as for any domain prediction tool, there exists the possibility that a domain is falsely predicted by CLADE. In this respect, several statistical tests to estimate CLADE false discovery rate (FDR) have been reported in [11], where it has been shown that, for the same FDR value, CLADE detects much more domains than its competitors, HMMer and HHblits. Run with default parameters (used for constructing Plasmobase), CLADE presents a FDR of 1*e*−3, that is, 1 in 1000 predictions are expected to be a false domain.

*Plasmobase contribution to new protein annotations* In Plasmobase, the user can explore and compare a large number of new domain annotations helping to the identification of a protein function. The protein PF3D7_1369500 illustrated in Fig. 1, for instance, contains three domains MIF4G/MIF4G-like obtained with E-value 1*e*−21, 1*e*−19 and 1*e*−8 and known to co-exist within protein sequences of metazoan, fungi and viridiplantae. In Plasmobase, this information, coming from UniProt, is accessible. The 96 UniProt metazoan sequences can be listed and their UniProt description can be looked up by clicking the protein accession number. By so doing, the user discovers that for these sequences, the architecture suggests the involvement of the protein in RNA metabolism and its role of RNA binding protein, but that its annotation goes from “uncharacterized protein” to “nuclear cap binding protein sub unit 1 (NCBP1)” inferred by homology, by experimental evidence at transcript level (in *Mustela pitorius furo* et *Xenopus laevis*) and by experimental evidence at protein level (in *Mus musculus* and *Homo sapiens*). This finding is supported by inspecting the 26 UniProt viridiplantae sequences. The majority of them was not studied but the *Arabidopsis thaliana* Q9SIU2 sequence was also annotated as NCBP1 by experimental evidence at protein level. From this analysis, one can infer that the annotation of NCBP1 is not solely a putative one because a biological validation was carried out in several studies conducted in mammals, *Xenopus* and *Arabidopsis*. Waiting for the biological validation in *Plasmodium*, this annotation could be proposed for the *Plasmodium* protein. This annotation is missing in PlasmoDB.

Another example is the PBANKA_0110700 protein with unknown function and no identified domain in Pfam. CLADE identified a peptidyl-tRNA hydrolase domain in it, with the extremely low E-value 2*e*−60.

Also, CLADE is helpful to confirm annotations identified with a low confidence. For instance, protein PF3D7_1452000 has no Pfam nor InterPro annotation but it is annotated as a rhoptry neck protein 2 (RON2) in PlasmoDB. In contrast, CLADE identifies the CLAG domain with an E-value 0 in PF3D7_1452000 based on a “clade-centred model” (in short CCM), that is a CLADE probabilistic model (see "Methods"). Note that homology between CLAG and RON2 has been previously assessed with a blastp search at E-value 0.001 [21], underlying the difficulty of current methods to identify divergent domains and the contribution of CCMs in raising the confidence.

*Plasmobase contribution to new protein architectures* The detection of new architectures is fundamental to the understanding of genome evolution. In this respect, DAMA was designed by trying to minimise the effect of prior knowledge on known architectures. This was done in two ways. First, DAMA combines information coming from different known architectures. This allows to identify new architectures with coexisting domains possibly belonging to different known ones. Second, DAMA searches for extra domains (not belonging to known architectures) that have no overlapping with those identified by exploiting known architectures, and that have a sufficient good score compared to the other predicted domains. These extra domains, satisfying the required conditions (overlapping and E-value), are added to the predicted architecture based on prior knowledge and enrich it. These two properties of the DAMA design assure the possibility to identify innovative architectures.

Plasmobase is a valuable new resource for domain annotation in *Plasmodium* genomes. All *Plasmodium* species reach a very large improvement in domain annotation, as reported in Table 3. Many *Plasmodium* sequences are also annotated for the first time. Plasmobase graphical presentation of protein sequences, based on predicted domain architectures, is of easy exploitation for comparative genomic studies. Plasmobase is expected to help to discover species-specific genes, possibly underlying important phenotypic differences between parasites, and orthologous gene families for deciphering the biology of these complex and important Apicomplexan organisms. The interactive nature of the platform allows the user to easily learn plenty of information on a given protein, on its protein family, on its orthologs in other *Plasmodium* species, and in other eukaryotes. This is especially important for those highly AT-rich proteins whose annotation by comparison to eukaryotic proteins is particularly difficult, due to sequence divergence.
